# The contribution of sensory nerves to cutaneous vasodilatation of the forearm and leg to local skin warming

**DOI:** 10.1186/2046-7648-4-S1-A125

**Published:** 2015-09-14

**Authors:** Matthew M Mallette, Gary J Hodges, Andrew T Del Pozzi, Gregory W McGarr, Stephen S Cheung

**Affiliations:** 1 Environmental Ergonomics Laboratory, Department of Kinesiology, Brock University, St. Catharines, Canada; 2 Departments of Pediatrics and Physiology, New York Medical College, Hawthrone, NY, USA

## Introduction

The initial cutaneous vasodilatory response to local skin heating is greater in the forearm than in the leg (1). While the initial vasodilatation of the forearm in response to local heating is primarily dependent on sensory nerves (2), their role in the leg is unknown. We compared the contribution of sensory nerves in regulating the cutaneous vasodilatory response of the forearm and leg to local heating using a local anaesthetic (EMLA) cream.

## Methods

In seven participants, two skin sites were selected on both the dorsal forearm and anterolateral calf; one site on each region received EMLA, while the other remained an untreated control. All sites were maintained at 33 °C and then locally heated to 42 °C at a rate of 1 °C·20 s^-1 ^with integrated laser-Doppler local heating probes. Finally, local heating to 44 °C was performed for 20 min to elicit maximal skin blood flow. Data are presented as cutaneous vascular conductance (CVC), calculated as laser-Doppler flux divided by mean arterial pressure from auscultation, and expressed as a percentage of maximal CVC.

## Results

At 33 °C forearm CVC was 8(3) %max at the untreated site and 5(2) %max at the EMLA treated site (*p *= 0.052). At the leg, CVC was 11(3) %max at the untreated site and 7(6) %max at the EMLA treated site (*p *= 0.298). There were no statistical differences for the untreated (*p *= 0.131) or the EMLA treated (*p=*0.202) skin sites between the forearm and leg. CVC during the initial vasodilatation to local heating was smaller in the leg (47(9) %max) compared to the forearm (62(7) %max, *p *= 0.012). EMLA reduced the initial vasodilatation at both the leg (27(13) %max, *p *= 0.02) and forearm (33(14)%max, *p*<0.001). The times to onset of vasodilatation, initial vasodilatory peak, and the plateau phase were longer in the leg compared to the forearm (all *p*<0.05), and EMLA increased these times in both regions (both *p*<0.05). CVC during the plateau phase to sustained local skin heating was higher in the leg compared to the forearm at both the untreated (93(6) *vs*. 85(4) %max, *p *= 0.33) and EMLA treated (94(5) *vs*. 86(6)%max, *p *= 0.001) sites; EMLA did not affect the plateau phase CVC on either limb (both *p *> 0.05).

## Discussion

Sensory nerve blockade with EMLA cream significantly decreased the initial vasodilatory response to local skin heating in both the forearm and leg. There were also marked differences in the temporal responses to local skin heating between the forearm and leg, with the onset of vasodilatation, initial vasodilatory peak, and establishment of the plateau slower to occur in the skin of the leg compared to the forearm. Interestingly, these limb differences in the initial peak and temporal responses were abolished under conditions of sensory nerve blockade.

## Conclusion

These data indicate that the differences in the initial vasodilatory response to local skin heating between the forearm and leg were due to sensory nerve involvement, as differences were abolished under conditions of sensory nerve blockade.
